# Nanoparticle-Based Delivery Strategies for Combating Drug Resistance in Cancer Therapeutics

**DOI:** 10.3390/cancers17162628

**Published:** 2025-08-11

**Authors:** Seohyun Park, Guo-Liang Lu, Yi-Chao Zheng, Emma K. Davison, Yan Li

**Affiliations:** 1 School of Science, Auckland University of Technology, Auckland 0102, New Zealand; 2 Auckland Cancer Society Research Centre, Faculty of Medical and Health Sciences, University of Auckland, Auckland 0102, New Zealand; 3 Maurice Wilkins Centre, University of Auckland, Auckland 0102, New Zealand; 4 State Key Laboratory of Metabolic Dysregulation & Prevention and Treatment of Esophageal Cancer, Zhengzhou University, Zhengzhou 450052, China; 5 Key Laboratory of Advanced Drug Preparation Technologies, Ministry of Education of China, Zhengzhou University, Zhengzhou 450001, China; 6 Key Laboratory of Henan Province for Drug Quality and Evaluation, School of Pharmaceutical Sciences, Zhengzhou University, Zhengzhou 450001, China

**Keywords:** Multidrug resistance (MDR), ABC transporters, nanoparticles, cancer therapeutics, nucleic acid-based therapies, cancer immunotherapy, drug delivery, chemoresistance, mononuclear phagocyte system, nanocarriers, efflux pumps, gene editing, stimuli-responsive delivery, CRISPR/Cas9 delivery, nanomedicine

## Abstract

Multidrug resistance (MDR) remains a formidable barrier to successful cancer treatment, driven by mechanisms such as efflux pump overexpression, enhanced DNA repair, evasion of apoptosis and the protective characteristics of the tumour microenvironment. Nanoparticle-based delivery systems have emerged as promising platforms capable of addressing these challenges by enhancing intracellular drug accumulation, enabling targeted delivery and facilitating stimuli-responsive and controlled release. This review provides a comprehensive overview of the molecular and cellular mechanisms underlying MDR and critically examines recent advances in nanoparticle strategies developed to overcome it. Various nanoparticle designs are analysed in terms of their structural and functional features, including surface modifications, active targeting ligands and responsiveness to tumour-specific cues. Particular emphasis is placed on the co-delivery of chemotherapeutic agents with gene regulators, such as siRNA, and the use of nanoparticles to deliver CRISPR/Cas9 gene editing tools as a means of re-sensitising resistant cancer cells. While significant progress has been made in preclinical settings, challenges such as tumour heterogeneity, limited clinical translation and immune clearance remain. Future directions include the integration of precision nanomedicine, scalable manufacturing and non-viral genome editing platforms. Collectively, nanoparticle-based drug delivery systems offer a multifaceted approach to combat MDR and hold great promise for improving therapeutic outcomes in resistant cancers.

## Simple Summary

Resistance to chemotherapy and targeted therapy remains one of the major obstacles in effective cancer treatment, often leading to poor therapeutic outcomes. This review explores how nanoparticle-based drug delivery systems can address this challenge by improving drug accumulation in tumours, enhancing targeting specificity and enabling controlled or stimulus-responsive drug release. In addition to conventional approaches, recent strategies involve co-delivery of chemotherapeutic agents with genome-editing tools to re-sensitise resistant cancer cells. By integrating emerging advances across multiple nanoparticle platforms, this review aims to provide a comprehensive understanding of their potential to overcome drug resistance. The findings discussed may inform future research and contribute to the development of more effective, personalised cancer therapies.

## 1. Introduction

Over the past several decades, significant progress has been made in the fight against cancer with the development of chemotherapeutic agents and targeted therapies, leading to improved overall survival rates [1]. However, cancer remains a major global health threat, accounting for approximately one in six deaths worldwide [2]. Despite huge advancements in anticancer therapies, chemotherapy still remains the most widely used treatment option for many cancer types, yet 90% of advanced/metastatic cancer patients eventually experience treatment failure, largely due to the emergence of multidrug resistance (MDR) in cancer cells [3,4,5,6].

MDR poses one of the most significant challenges in cancer treatment, as it severely undermines the efficacy of existing therapies by enhancing tumour cell resistance to a broad range of chemotherapeutic agents, particularly after prolonged drug exposure [7]. Tumour cells employ various intrinsic and acquired mechanisms to evade the cytotoxic effects of chemotherapeutic agents. Intrinsic resistance exists before the initiation of therapy and is affected by factors such as age, sex and genetic predisposition, while acquired resistance develops over the course of treatment as a result of various therapy-induced adaptive responses [8,9].

To address this major challenge, many strategies have been explored, and particularly, nanoparticle (NP)-based drug delivery systems have emerged as a promising strategy [3]. NPs can be engineered to encapsulate efflux pump inhibitors, which are known to play a crucial role in overcoming cancer MDR by preventing the active efflux of chemotherapeutic agents from cancer cells [4,10]. The structural versatility, easily modifiable surface properties and nanoscale sizes of NPs allow the co-delivery of multiple chemotherapeutic agents, facilitating combination therapy approaches using a single platform [11,12]. Additionally, surface modification with different ligands or antibodies can enhance targeted therapy by recognising tumour-specific biomarkers, to improve tumour-selective accumulation [13]. Furthermore, the multifunctionality of NPs enables them to encapsulate and deliver gene-editing tools such as small interfering RNA (siRNA) and clustered regularly interspaced short palindromic repeat (CRISPR)/Cas9 components, offering the potential to directly modulate the expression of genes associated with drug resistance [14]. NP-based drug delivery systems also improve the pharmacokinetics of chemotherapeutics by prolonging circulation and enhancing tumour accumulation [15]. In fact, polymer-based nanocarriers have been shown to facilitate selective tumour targeting, extend drug half-life and provide controlled release of drugs, thereby increasing the effective drug concentration at resistant tumour sites [16]. These features simultaneously reduce off-target toxicity, addressing one of the primary drawbacks of conventional chemotherapy [15].

Recent reviews have provided a comprehensive survey of lipid- and polymer-based platforms for efflux-pump inhibition, and some have focused on CRISPR/Cas9 delivery via NPs to knock out resistance genes [17,18]. Additionally, NP-based approaches to inhibiting key resistance pathways were reviewed and noted that nanocarriers can achieve coordinated pharmacokinetic/pharmacodynamic profiles by co-encapsulating drugs with resistance modulators [15]. More recently, it was found that multi-drug NP formulations significantly outperform both single-agent and free-drug combinations in resistant tumour models [19]. However, no review has comprehensively integrated pump-based inhibitors, non-pump mechanisms and gene-editing strategies into a single cohesive framework. Here, we present an up-to-date overview of NP-based tactics to reverse or bypass drug resistance in cancer therapy—discussing recent advances and highlighting their promise for improving therapeutic outcomes. Nevertheless, translating NP-based strategies from bench to bedside remains challenging due to issues like tumour heterogeneity, potential nanocarrier immunogenicity and manufacturing constraints [16]. This review also aims to briefly discuss these hurdles and potential solutions in the context of developing clinically viable MDR therapies.

## 2. Mechanisms of Drug Resistance in Cancer

MDR in cancer cells arises through several mechanisms, including enhanced drug efflux via membrane-bound efflux pumps, alterations in drug targets, enhanced DNA repair mechanisms, evasion of apoptosis and modification of the tumour microenvironment (TME) [7,20,21]. A comprehensive understanding of these resistance mechanisms is important for the rational design of NP-based strategies aimed at overcoming MDR and improving therapeutic efficacy.

Intrinsic and acquired resistance share some common resistance mechanisms and increase in drug efflux, enhanced DNA damage repair, alterations in expression levels or mutation of drug targets, alterations in drug metabolism, epigenetic modifications, activation of alternative compensatory signalling pathways, activation of survival signalling pathways and inactivation of downstream death signalling pathways are some mechanisms in which resistance in tumours may occur [22,23,24,25]. Furthermore, tumours often exhibit significant molecular heterogeneity, and as a result, a substantial contribution to drug resistance can develop through the therapy-induced selection of a minor subpopulation of resistant cells (from the original tumour) [23,26]. Systemic toxicity often limits the maximum dose that can be safely administered, resulting in sub-lethal drug levels at the tumour. This under-dosing imposes a strong selection pressure—effective on sensitive cells while allowing partially resistant clones to survive and proliferate, thereby promoting MDR development [23].

### 2.1. ABC Transporters and Multidrug Resistance

**Figure 1:**
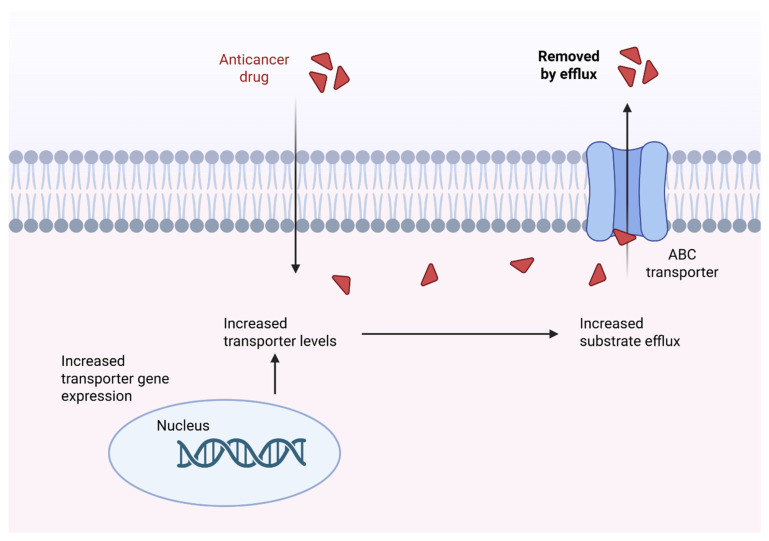
General mechanism of drug efflux via ABC transporters. ABC transporters actively pump chemotherapeutic agents across the plasma membrane, reducing the intracellular accumulation. This ATP-dependent process limits anticancer drugs from reaching therapeutic levels within the tumour cells, leading to MDR. This figure was created in BioRender (Park, S. (2025); https://BioRender.com/lk05ph5, accessed on 13 May 2025).

Efflux transporter-mediated drug resistance is one of the most well-characterised mechanisms. The human ATP-binding cassette (ABC) transporter superfamily consists of seven families and 48 individual family members, many of which function to recognise substrate molecules (e.g., drugs, lipids, metabolites and toxins) and use energy from ATP hydrolysis to translocate these molecules across cell membranes [22,27,28]. The most extensively studied functions of these transporters include their role in MDR in cancer, detoxification and protection against xenobiotics, lipid and cholesterol transport, and antigen presentation and immune modulation [27,29]. ABCB1 (P-gp/P-glycoprotein ABCB1), multidrug resistance-associated proteins (MRPs/ABCC family) and breast cancer resistance protein (BCRP/ABCG2) have been widely implicated in the development of drug resistance across various cancer types [30]. The most well-understood role of ABC transporters is in MDR; they cause active efflux of a wide range chemotherapeutic drugs from cancer cells, thereby reducing intracellular drug accumulation, which limits their therapeutic efficacy (Figure 1) [3,30]. Previous studies have demonstrated that prolonged administration of chemotherapeutic agents may increase the expression of ABC transporters, consequently increasing the risk of MDR [31,32]. Drug transporters significantly influence the pharmacokinetics, efficacy and toxicity derived from therapeutic agents [33]. Moreover, transporter-mediated drug−drug interactions are common, as many therapeutic agents act as substrates or inhibitors of transporter proteins [33].

#### 2.1.1. ABCB1(P-Glycoprotein/P-gp)

ABCB1, a 170 kDa transporter expressed in the liver, intestine and brain, confers resistance to various chemotherapeutics, including taxanes, vinca alkaloids and anthracyclines [34]. Its overexpression in cancer cells has been linked to significant limitations in the intracellular drug accumulation [34]. Although multiple generations of P-gp inhibitors have been developed, none are currently approved for clinical use due to toxicity and pharmacokinetic limitations [34]. Previous attempts were made to develop ABCB1 inhibitors for clinical use to overcome multidrug resistance, but they failed in clinical trials primarily because the trials did not limit patient selection to those whose tumours were nonresponsive to treatment due to ABC transporter overexpression [28,35]. Nonetheless, some third- and fourth-generation modulators (e.g., Tariquidar, Elacridar and Curcumin) are promising [36]. NP-based co-therapeutics targeting P-gp are under development to enhance treatment efficacy in resistant tumours [37].

#### 2.1.2. ABCG2 (Breast Cancer Resistance Protein)

BCRP, a half-transporter, reduces intracellular accumulation of agents such as topotecan, irinotecan, mitoxantrone and various tyrosine kinase inhibitors (TKIs) [33,38,39]. Its broad substrate specificity and high expression in cancer stem cells further complicate treatment outcomes and contribute to relapse and metastasis by protecting cancer cells from chemotherapeutic agents [40]. Inhibitors like Ko143 and SCO-101 have demonstrated preclinical success in reversing BCRP-mediated resistance; however, clinical translation remains limited due to specificity, toxicity and pharmacokinetic interactions. Ongoing efforts are exploring NP-based systems to bypass or competitively inhibit BCRP activity [39,41].

#### 2.1.3. ABCC Family Members (Multidrug Resistance-Associated Proteins)

MRP2 (ABCC2) is highly expressed in the gastrointestinal (GI) system and has been closely linked to resistance in GI cancers, particularly to platinum-based drugs [42,43]. It is an active transporter that uses energy from ATP hydrolysis to transport substrates across cell membranes [44]. MRP2 has been found to actively transport chemotherapeutic agents into intracellular vesicles or out of cells, contributing to chemoresistance. A clinical study in metastatic colorectal cancer (CRC) patients demonstrated significantly higher tumour MRP2 expression in non-responders to oxaliplatin-based combination chemotherapy, correlating to elevated ABCC2 mRNA with reduced intracellular drug accumulation and oxaliplatin resistance [42]. Functional studies have shown that suppression of the functional expression of MRP2, by siRNA gene knockdown or myricetin (a model MRP2 inhibitor), reversed those deficits in oxaliplatin accumulation and increased oxaliplatin antitumour cytotoxicity. A study of mice bearing tumour xenografts of a human GI tumour line with endogenous MRP2 overexpression demonstrated that inhibiting MRP2 with myricetin increased in vivo sensitisation to oxaliplatin antitumour activity with little or no increase in host toxicity. These findings are consistent with reports from elsewhere of experimental [45,46] and clinical studies [47,48,49] on the influence of ABCC2 on oxaliplatin-based therapy of GI cancer.

MRP5 (ABCC5) is a ~190 kDa ABC transporter, broadly expressed on the basolateral membrane of epithelial cells in liver, kidney and pancreas, as well as in endothelial and certain immune cells. Similarly, it contributes to resistance against nucleoside analogues, particularly gemcitabine, in pancreatic ductal adenocarcinoma (PDAC) [50]. Knockdown of MRP5 in MIA PaCa-2 and PANC-1 cells using lentiviral shRNA decreased MRP5 mRNA and protein expression, resulting in enhanced gemcitabine sensitivity, increased BCECF accumulation and elevated caspase-3/7-mediated apoptosis [50]. This effect was not observed with non-MRP5 substrates like olaparib, confirming its substrate specificity. MRP5 silencing also impaired colony formation, wound healing and migration, suggesting pro-tumourigenic roles beyond drug transport. These effects may be partially explained by MRP5-mediated cyclic GMP (cGMP) efflux, as suggested by in silico docking studies [50]. To date, MRP5 knockdown by lentiviral shRNA remains at the pre-clinical stage—limited to in vitro and xenograft models in cell lines such as MIA PaCa-2 and PANC-1—and no MRP5-specific small-molecule inhibitor has advanced into human studies [50]. Broad-spectrum ABCC inhibitors such as MK571 and probenecid can inhibit MRP5 activity in vitro, but they lack selectivity and are not clinically approved for MDR reversal [51].

### 2.2. Beyond Drug Efflux: Mechanistic Roles of ABC Transporters in Cancer Resistance

**Figure 2:**
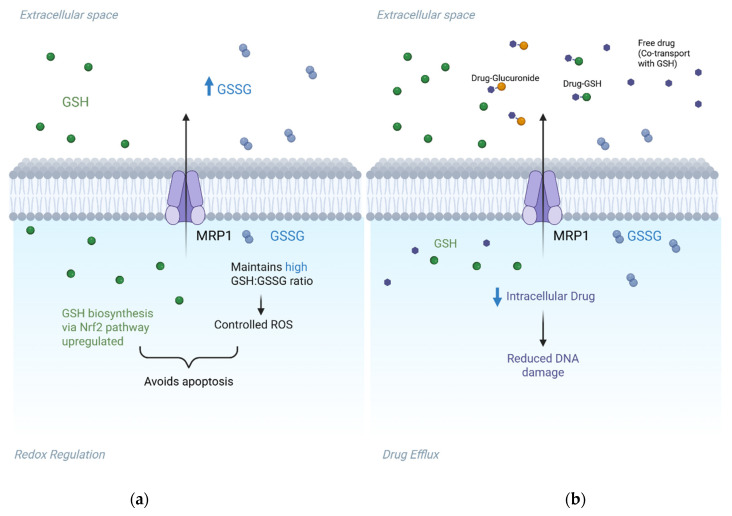
MRP1 supports multidrug resistance by maintaining the following: (**a**) redox balance through GSSG efflux; (**b**) exporting GSH-conjugated drugs to reduce intracellular drug levels and DNA damage. This figure was created in BioRender (Park, S. (2025); https://BioRender.com/pynh1p9, accessed on 13 May 2025.

**Figure 3:**
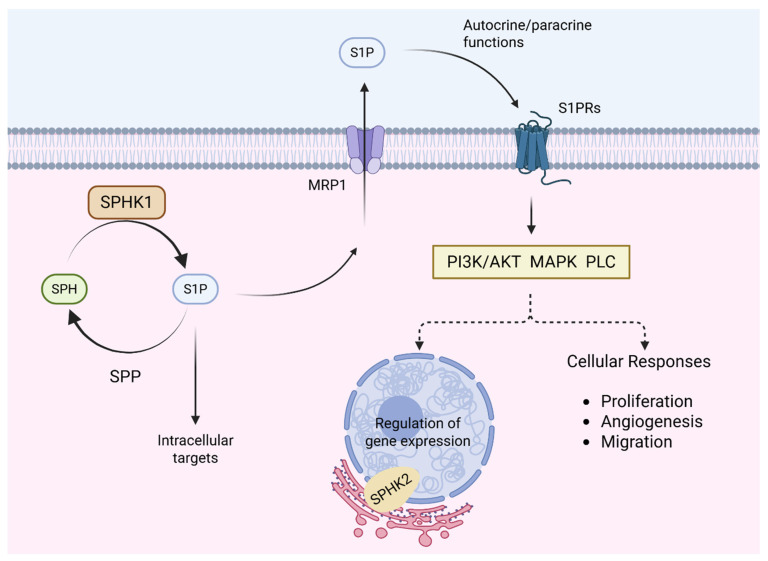
MRP1-mediated S1P export and downstream signalling. S1P, generated from sphingosine by SPHK1, can act on intracellular targets or be exported from the cell by MRP1. Extracellular S1P binds to sphingosine-1-phosphate receptors (S1PRs) in an autocrine or paracrine manner, activating PI3K/AKT, MAPK, and PLC pathways. This signalling cascade regulates gene expression via SPHK2 and promotes cellular processes such as proliferation, angiogenesis, and migration. This figure was created in BioRender (Park, S. (2025); https://BioRender.com/da7ybu9, accessed on 13 May 2025).

Beyond their well-characterised role in drug efflux, ABC transporters contribute to cancer resistance through diverse non-canonical mechanisms that support tumour survival and adaptability. ABCB1, ABCC2 and ABCG2 help limit DNA damage by exporting carcinogens such as PhIP and NNK, while ABCB7 supports genomic integrity through the transport of iron-sulphur cluster precursors essential for DNA repair [52]. MRP1 (ABCC1) maintains redox homeostasis by exporting reduced and oxidised glutathione (GSH) to preserve the intracellular GSH:GSSG ratio, a key defence against chemotherapy-induced oxidative stress (Figure 2) [9,53,54,55]. Under chemotherapy-induced oxidative stress, this antioxidant defence mechanism allows cancer cells to suppress reactive oxygen species (ROS) accumulation, evade apoptosis and develop chemoresistance [56]. ABCG2 and ABCB7 further contribute to ROS regulation through antioxidant signalling and mitochondrial iron control [52,57,58]. Tumour microenvironmental cues can also modulate transporter function post-translationally. In CRC, tumour-associated macrophages secrete CCL17 and CCL22, which do not increase total MRP1 levels but instead trigger its relocalisation from intracellular stores to the plasma membrane. This results in enhanced MRP1-mediated efflux of 5-fluorouracil without any change in MRP1 expression [59]. (Here, CCL17/22 engagement activates the PI3K/AKT pathway—lipid-kinase/serine-threonine-kinase cascade that, via the chaperone GRP78, drives MRP1 trafficking to the cell surface.) ABC transporters also drive tumour vascularisation. MRP1-mediated sphingosine-1-phosphate (S1P) export activates SPHK1 and promotes angiogenesis (Figure 3), while ABCA1 externalises calpain to facilitate endothelial migration and ABCB1/ABCC4 regulate capillary morphogenesis [60,61,62]. In metabolism, ABCC3 sustains glycolytic activity in bladder cancer by modulating lactate dehydrogenase A expression, with its knockdown impairing glycolysis and sensitising cells to cisplatin [63]. Immune evasion is further supported by reduced TAP1/2 expression, which disrupts MHC class I antigen presentation and impairs CD8^+^ T cell recognition, while ABCA1 promotes M2 macrophage polarisation via 27-hydroxycholesterol, fostering immunosuppression [64]. ABC transporters have also been implicated in epigenetic and transcriptional regulation; ABCC7 modulates miR-193b and tumour suppressor expression in prostate cancer, while ABCG2 transports micronutrients like folate that serve as cofactors for methylation reactions affecting chromatin structure and gene expression [65,66,67]. These emerging roles highlight how ABC transporters modulate redox signalling, immune escape, angiogenesis and epigenetic modulation, reinforcing their significance as multidimensional drivers of resistance beyond drug efflux.

### 2.3. Other Mechanisms

#### 2.3.1. Alterations in Drug Targets

The therapeutic efficacy of anticancer drugs depends on their ability to bind specific molecular targets. However, mutations or changes in the expression levels of these targets can compromise treatment outcomes. For example, resistance to topoisomerase II-targeting drugs like doxorubicin can develop due to mutations in the topoisomerase II gene, which hinder effective drug−target interactions [68]. These mutations prevent the drug from stabilising the topoisomerase II-DNA complex, thus reducing its ability to induce DNA damage and cell death. Similarly, changes in the expression of targets like epidermal growth factor receptor (EGFR) in non-small cell lung cancer can lead to resistance to EGFR inhibitors, as mutations or overexpression can alter drug binding, rendering the therapy less effective [69].

#### 2.3.2. Enhanced DNA Repair Mechanisms

Many chemotherapeutic agents exert their effects by inducing DNA damage in tumour cells. For example, cisplatin, commonly used for the treatment of lung adenocarcinoma, induces double-strand breaks in tumour cells that ultimately trigger cell death. However, lung cancer cells can often upregulate their DNA repair pathways to overcome cisplatin-derived toxicity [70]. This includes enhanced activity of nucleotide excision repair pathways and homologous recombination repair mechanisms, which efficiently repair drug-induced DNA lesions and contribute to drug resistance.

#### 2.3.3. Evasion of Apoptosis

Evasion of apoptosis is a well-recognised hallmark of cancer and plays a critical role in MDR. Cancer cells often achieve this through the overexpression of anti-apoptotic proteins such as BCL2 or the downregulation of pro-apoptotic proteins, making them resistant to anticancer drugs that rely on apoptosis induction for their cytotoxic effects [71]. Dysregulation of apoptotic signalling pathways enables tumour cells to evade programmed cell death even in the presence of chemotherapeutic agents.

#### 2.3.4. Tumour Microenvironment Barriers

The TME represents one of the early barriers that therapeutic agents must overcome to achieve effective tumour targeting. Characterised by hypoxia, acidic pH, a dense extracellular matrix, elevated interstitial fluid pressure and abnormal vasculature, the TME’s complex architecture contributes to the development of TME-induced clinical resistance [72]. Among the cellular components of the TME, cancer-associated fibroblasts play a key role by secreting pro-tumourigenic factors such as transforming growth factor-beta, vascular endothelial growth factor and fibroblast growth factor-2, which promote metastasis and therapeutic resistance in CRC cell lines [72]. Furthermore, hypoxia-inducible factor-1 (HIF-1) allows cancer cells to adapt to hypoxic conditions within the TME by regulating the expression of multiple cancer stem cell markers, thereby enhancing tumour cell survival and reducing drug sensitivity [73].

## 3. Nanoparticle-Based Drug Delivery Systems: An Overview

The current challenges associated with conventional cancer therapeutics include off-target toxicity, low specificity and limitations on the maximum dose that can be safely administered due to severe systemic side effects [74,75]. As a result, the therapeutic window for many anticancer drugs remains narrow, restricting their clinical efficacy and contributing to suboptimal patient outcomes [74,75]. Moreover, the long-term efficacy of these treatments is often compromised by the development of MDR in cancer cells, which remains a significant barrier to cancer treatment [76].

NP-based drug delivery systems have emerged as a promising strategy to address these limitations. By enhancing tumour-selective drug accumulation and minimising systemic toxicity, NPs improve therapeutic efficacy and reduce off-target effects [77]. NPs as drug delivery systems must exhibit good biocompatibility and biodegradability, demonstrate stability in physiological conditions and possess the ability to carry a high drug payload with minimal toxicity to ensure safe and effective treatment [78]. Their ability to increase intracellular drug concentration at the tumour site occurs via multiple mechanisms, including passive targeting through the enhanced permeability and retention (EPR) effect, and active targeting via surface modification with ligands or antibodies [75,79].

### 3.1. Nanoparticles in Cancer Therapy

A wide range of NPs have been explored for drug delivery in cancer research, each offering unique physicochemical properties that can be tailored to combat drug resistance. Specific sizes, shapes and surface characteristics can significantly impact the efficiency of nano-drug delivery and thereby impact the therapeutic efficacy. Among these, lipid-based NPs, polymer-based NPs, inorganic NPs and even hybrid NPs have shown considerable potential in both preclinical and clinical studies [80]. Lipid-based NPs, including liposomes, solid lipid nanoparticles (SLNs), nanostructure lipid carriers and lipid nanoparticles (LNPs) used clinically in mRNA vaccines and siRNA therapies, represent a broad and versatile class of nanocarriers [81,82,83,84,85,86]. Liposomes, composed of phospholipid bilayers surrounding an aqueous core, are among the most clinically established NPs for targeted drug delivery, with several U.S. Food and Drug Administration (FDA)-approved formulations such as Doxil^®^ for ovarian cancer (Table 1) [87,88]. The biocompatibility, ability to encapsulate both hydrophilic and hydrophobic drugs and potential for surface modification of liposomes make them ideal candidates as drug delivery systems to overcome drug resistance in cancer therapy.

In addition to liposomes, other nanocarrier platforms such as polymeric and inorganic NPs have demonstrated considerable potential in overcoming drug resistance in cancer therapy [16]. Polymeric NPs, including micelles and dendrimers, offer tuneable size, surface functionality and controlled drug release profiles. For example, polymeric micelles formed from amphiphilic block copolymers have been employed to deliver hydrophobic chemotherapeutic agents and overcome P-gp-mediated resistance by enabling intracellular accumulation through endocytosis pathways [97]. Dendrimers, due to their highly branched architecture and multivalency, also allow for the co-delivery of drugs and nucleic acids, improving therapeutic synergy against resistant tumour cells [98]. Inorganic NPs, such as gold nanoshells, quantum dots and iron oxide NPs, offer unique optical, magnetic and structural properties that facilitate both drug delivery and imaging [99]. Notably, gold NPs have been used for photothermal ablation of resistant tumours, while iron oxide particles aid in magnetic-targeted delivery and real-time tracking [100]. Though these systems are at varying stages of clinical translation, they complement lipid-based carriers and further broaden the landscape of NP-mediated approaches to circumvent multidrug resistance.

**Figure 4:**
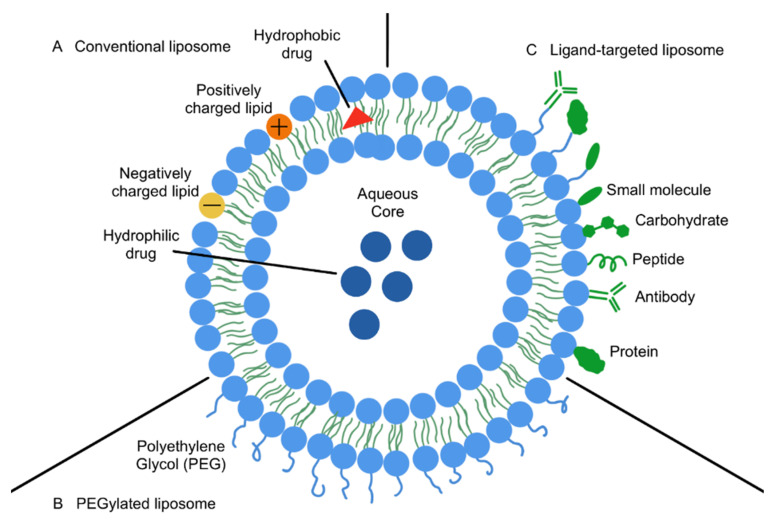
Common liposome surface modifications.

Conventional liposomes can be synthesised with or without cholesterol, where cholesterol incorporation provides stability and increases membrane ordering [87]. However, conventional liposomes are prone to rapid clearance from the bloodstream, resulting in shortened circulation times and limited intracellular drug availability at the target site. To overcome these limitations, advanced liposomal formulations have been developed, including active targeting liposomes, stimuli-responsive liposomes and stealth liposomes modified with polyethylene glycol (PEG) to prolong circulation time and evade immune detection (Figure 4). Different types of NP platforms are summarised in Table 2 below.

### 3.2. Strategies to Minimise Immune Clearance

Even with advanced “stealth” nanomedicine formulations, around 99% of the systemically administered PEGylated nanoparticles end up accumulating in the mononuclear phagocyte system (MPS) and other non-target tissues [114]. Uptake by MPS plays a pivotal role in the clearance of systemically administered nanoparticles [115], contributing to the insufficient tumour accumulation and limited clinical efficacy. Use of immune-evasive polymers beyond PEG, such as zwitterionic materials (e.g., poly(carboxybetaine) lipids), is gaining traction as an effective strategy to mitigate the accelerated blood clearance effect [116]. Emerging evidence also suggests nanoparticle-triggered immune responses can be harnessed to decrease off-target uptake while enhancing tumour accumulation in mice. Pretreatment with interferon lambda (IFN-λ) or lipoplexes triggers an innate immune response that reduces the off-target accumulation of subsequently administered Doxil^®^ [117], highlighting a novel strategy to tumour-targeted delivery of nanomedicines. Delivery of anticancer nanomedicines may be further hindered by the immunosuppressive tumour microenvironment, including tumour-associated macrophages (TAMs), regulatory T cells (Tregs) and myeloid-derived suppressor cells (MDSCs) [118,119,120,121,122]. To reverse TME dysfunction, delivering immunomodulatory molecules (e.g., alendronate [120] and a stimulator of interferon genes (STING) agonist [123]) via PEGylated nanocarriers can be exploited to activate cytotoxic T-cells or NK cells and increase antitumour cytokine production in mice. Given the species differences in immunological interactions, pharmacokinetics and pharmacodynamics, more clinically relevant in vivo tools (e.g., humanised animal models) are needed to facilitate the translation of personalised anticancer nanomedicines [124].

## 4. Nanoparticle Strategies to Overcome Multidrug Resistance

Structural modifications of NPs, including surface conjugation with targeting ligands, incorporation of efflux pump inhibitors or integration of gene-editing components, to name a few, have emerged as promising strategies to overcome drug resistance in cancer therapy [125]. Some of the major mechanisms of MDR can be overcome through the engineering of NPs with specific design features. These include passive targeting via the EPR effect and active targeting through surface conjugation of various ligands, including antibodies and stimuli-responsive NPs [125].

### 4.1. Enhanced Tumour Accumulation (The EPR Effect)

**Figure 5:**
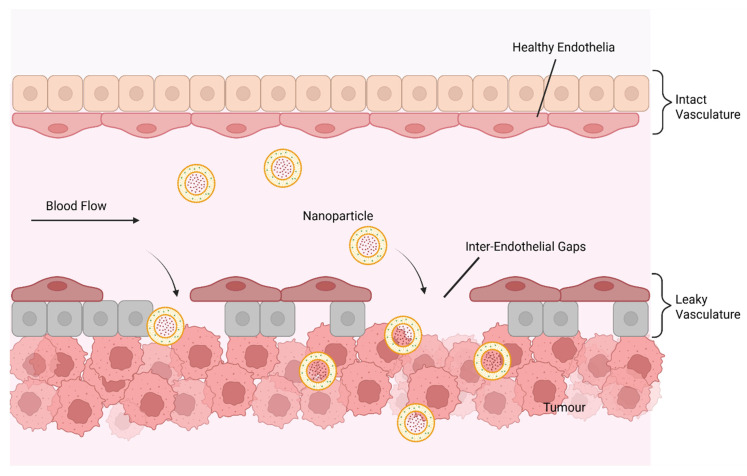
Schematic illustration of the enhanced permeability and retention effect. NPs circulate through the bloodstream and preferentially accumulate in tumour tissues due to the presence of inter-endothelial gaps in the abnormal, leaky tumour vasculature. Upon entering the tumour microenvironment, their clearance is hindered due to poor lymphatic drainage, resulting in prolonged retention and increased local drug concentration. This figure was created in BioRender (Park, S. (2025); https://BioRender.com/r0xlwkg, accessed on 13 May 2025).

The EPR effect is a phenomenon where NPs preferentially accumulate in tumour tissues through inter-endothelial gaps due to leaky vasculature and are unable to exit efficiently due to poor lymphatic drainage (Figure 5) [125]. Blood vessels with compromised endothelial cells emerge as tumours rapidly grow and require significant amounts of oxygen and other nutrients [126]. The design features of NPs directly influence how well they exploit the EPR effect, including size, surface charge, hydrophilic coatings, shape, stability in circulation and passive targeting via leaky vasculature (Table 3) [127,128,129,130]. EPR-based tumour targeting, however, is not applicable to all tumours due to nonspecific distribution, insufficient tumour accumulation, high interstitial fluid pressure, hypoxia and other tumour heterogeneity that leads to variations.

Additionally, more recent studies have highlighted another potential mechanism of NP entry—the active transport and retention (ATR) principle [131]. This principle suggests that NPs primarily enter tumour tissues through an active process such as transcytosis mediated by NP transport endothelial cells and are subsequently retained due to interactions with tumour cellular receptors and the extracellular matrix (ECM) components. For example, liposomal and silica NPs have been shown to bind to tumour-associated collagens I and IV, fibronectin and hyaluronic acid with the interstitial matrix, while engagement of cell-surface lectins (e.g., galectin-3) and integrins (e.g., ανβ3) further anchors them to cancer and stromal cells [125,131,132]. In fact, a recent study revealed that up to 97% of gold, silica and liposome NPs accumulated via these active transport and the ECM/receptor-mediated retention pathways in murine breast tumour models [132]. However, the ATR principle has so far only been characterised for gold, silica and liposomal formulations and is yet to be elucidated in other types of NPs [67].

### 4.2. Active Targeting via Surface Conjugation

Active targeting strategies involve modifying the surface of NPs with tumour-specific ligands that can recognise and bind to overexpressed receptors on the surface of cancer cells [18]. This receptor−ligand interaction facilitates receptor-mediated endocytosis, leading to enhanced internalisation of the NP and its therapeutic payload by the target cells, thereby improving drug accumulation at the target site while reducing off-target effects [133]. There are several active targeting NPs that have been studied for cancer therapy with different targets (Table 4). A wide range of targeting moieties is available, including monoclonal antibodies, antibody fragments, peptides, aptamers, hyaluronic acid and small molecules such as folic acid [134,135].

#### 4.2.1. Folate Conjugation

Folate-functionalised liposomes have shown promise in targeting folate receptor-overexpressing tumours, particularly in ovarian and breast cancers [160]. A recent study demonstrated a significant increase in intracellular drug delivery and enhanced antitumour activity in CRC using folate-conjugated pH-sensitive liposomes (PSLs) for the delivery of irinotecan as opposed to the free drug or pH-responsive system only [161].

#### 4.2.2. RGD Peptide Conjugation

Similarly, another promising strategy to overcome MDR and enhance therapy efficacy, especially in breast cancer, is the development of arginine-glycine-aspartic (RGD) peptide-modified, pH-responsive solid lipid NPs (RGD-DOX-SLNs). This approach leverages the high expression of integrin αvβ3 on tumour neovasculature, which is a common feature in breast cancer, to target NPs specifically to the tumour site. The RGD peptide is conjugated to NPs to ensure effective targeting to αvβ3 integrins, which are found abundantly on the surface of tumour-associated endothelial cells [148].

To further enhance the specificity and effectiveness, these RGD-DOX-SLNs are modified with pH-sensitive lipids that facilitate controlled release of their cargo in acidic environments of the TME. The pH-sensitive linker ensures that the NPs remain stable and intact in the bloodstream and upon acidification in the tumours they release the encapsulated drug such as doxorubicin. This targeted release minimises exposure of healthy tissues to the chemotherapy, thereby reducing systemic toxicity [148].

The RGD-DOX-SLNs are 96.3 nm with a narrow size distribution and a zeta potential of 35.6 mV, a high surface charge that provides strong electrostatic repulsion and thus contributes to their colloidal stability. They demonstrated a drug loading of 9.8%, and the resulting encapsulation efficiency was 98.5%. The in vitro release profile at pH 5.0 followed a biphasic pattern whereby a rapid initial burst was followed by slow, sustained release until the 96 h endpoint of the study. At physiological pH, however, the drug release rate demonstrated slightly lower release patterns. RGD-DOX-SLNs demonstrated enhanced cytotoxicity compared to non-modified DOX-SLNs. Compared to free doxorubicin, RGD-DOX-SLNs exhibited a 5.58-fold higher area under the plasma concentration-time curve and significantly higher tumour growth inhibition in in vivo models [148].

#### 4.2.3. Aptamer-Functionalised NPs

Aptamers are short, single-stranded oligonucleotides that can specifically bind to cell surface markers or receptors. Aptamers offer several advantages due to their small size, low immunogenicity and ease of chemical modification, which improve safety, specificity, biodistribution and organ accumulation. Their abilities to penetrate tissues better, reduce immune responses and be chemically engineered for enhanced stability and pharmacokinetics, along with their potential to target non-immunogenic proteins, make aptamers highly versatile for targeted therapeutic delivery [162]. In previous studies, aptamer-conjugated NPs targeting overexpressed receptors like PD-L1 or prostate-specific membrane antigen (PSMA) have demonstrated high specificity and reduced off-target effects. For instance, aptamers targeting PD-L1 have been utilised to deliver siRNA therapeutics, effectively silencing PD-L1 expression and enhancing antitumour immunity in triple-negative breast cancer cells. Similarly, PSMA-targeted aptamers, such as A10, have been conjugated with NPs to deliver chemotherapeutic agents like doxorubicin specifically to prostate cancer cells, resulting in increased drug accumulation at the tumour site and reduced systemic toxicity [162,163].

#### 4.2.4. Transferrin-Receptor-Targeted NPs

Transferrin (Tf), the receptor for iron uptake, is overexpressed in many cancer cells, including those with MDR [138]. Tf-conjugated NPs that deliver drugs like doxorubicin have shown targeted delivery to drug-resistant cells that actively take up transferrin and its conjugates [138]. This strategy is also explored for the treatment of glioblastoma as the Tf receptor is abundantly distributed in glioblastoma and blood−brain barrier cells, and many studies have developed transferrin-modified nanosystems for glioblastoma therapy [139].

#### 4.2.5. CD44-Targeted NPs

CD44, a cell surface glycoprotein involved in cell−cell interactions, is overexpressed in many cancers such as pancreatic, breast, ovarian, brain and lung cancers, compared to normal cells [164]. Its role in targeting tumour endothelial cells has been established previously, highlighting its importance in overall tumour targeting [165]. Many studies have demonstrated NP systems conjugated with hyaluronic acid, a CD44 native ligand, as a promising approach to the targeted delivery of therapeutic agents to cancer stem cells, which are often responsible for MDR [164,166].

#### 4.2.6. Monoclonal Antibody-Conjugated NPs

Monoclonal antibodies (mAbs) that target specific tumour antigens are increasingly being used in NP-based drug delivery systems. Due to their higher binding affinity and specificity, mAbs are increasingly studied for selective cancer biomarker detection and treatment [167]. These can be conjugated to NPs to enhance the specificity and selectivity of the treatment, ensuring that the encapsulated agent is directed precisely to tumour tissues while minimising off-target effects.

In recent studies, Trastuzumab-conjugated paclitaxel-loaded nanorods (PTXNR-TTZ) demonstrated a significant increase in therapeutic efficacy for HER2-positive breast cancer. They exhibited enhanced cellular uptake and superior cytotoxicity compared to free drugs or single-agent treatments. Particularly, PTXNR-TTZ inhibited BT-474 and SK-BR-3 HER2-positive cells by more than 80%, showing synergistic effects in comparison to individual treatments of either paclitaxel or trastuzumab alone. Moreover, a cell cycle arrest in the G2/M phase of the cell cycle, a key pathway for apoptosis induction, was observed. This was further supported by caspase-dependent apoptosis, confirming the induction of cell death, with cleaved caspase-9, caspase-3 and cytochrome C levels significantly increasing, while the anti-apoptotic XIAP was downregulated. This approach, combining the specificity of monoclonal antibodies with the controlled release capabilities of NPs, demonstrates a promising strategy to address challenges in MDR [168].

Programmed death-1 (PD-1) is an immune checkpoint receptor expressed on T cells that suppresses immune activation and promotes tumour immune evasion upon binding to PD-L1 on tumour cells [169]. A previous study has demonstrated that docetaxel-loaded PEG-PCL NPs conjugated with PD-L1 mAbs enhance uptake in gastric cancer cells that overexpress PD-L1. An increase in apoptosis, G2/M cell cycle arrest and disruption in microtubule dynamics were observed, as well as sustained drug release and targeted delivery compared to the non-conjugated NPs [169]. Notably, this system also synergised with docetaxel’s cytotoxic activity, potentially overcoming immune evasion mechanisms [169]. This study highlighted the potential of mAb-conjugated NPs to optimise chemotherapeutic agent delivery while simultaneously modulating the tumour immune environment.

#### 4.2.7. Dynamic Surface Engineering for Tumour Heterogeneity

To address tumour heterogeneity and inconsistent NP uptake by tumours, recent strategies have focused on dynamic surface modifications. Charge-reversal systems are one promising approach where NPs are designed to remain neutral or anionic in circulation and become cationic within the TME, in response to tumour-specific enzymes such as γ-glutamyl transferase [170]. This enables them to shift from a “stealth” state to a cell-adhesive form within the TME, resulting in enhanced cellular uptake and tumour retention [170]. pH- and enzyme-responsive coatings similarly enhance intratumoural delivery by activating in acidic or protease-rich conditions which is discussed further in the following subsection [171]. Zwitterionic surfaces, such as those based on phosphorylcholine, reduce protein corona formation and improve the consistency of biodistribution. In addition, targeting ligands that remain masked during circulation and then revealed only near the tumour site have exhibited better specificity [172]. Taken together, these adaptable surface designs help overcome biological variability and enhance the precision of NP-based therapies in resistant tumours.

### 4.3. Stimuli-Responsive Nanoparticles

Stimuli-responsive NPs are engineered to release therapeutic cargo in response to specific triggers within the TME or via externally applied stimuli [18]. These triggers can range from pH alterations, redox potential, enzyme activity, hypoxia or application of different physical stimuli (Table 5). Stimuli-responsive NPs respond to these tumour-associated cues and achieve controlled drug release, enhanced tumour selectivity and improved efficacy in overcoming MDR [173,174].

#### 4.3.1. Metabolic Targeting via Lactate-Responsive NPs for Tumour-Specific Drug Release

More recently, a novel active targeting approach involves metabolic targeting, wherein the surface of NPs is functionalised to respond to specific metabolic alterations in tumours, such as the Warburg effect, where there are elevated lactate levels in the TME [183]. NPs engineered with lactate oxidase and arylboronate gating derivatives demonstrated release of their cargo in response to elevated lactate levels in tumour tissues. The mechanism underlying this design involves the conversion of lactate to hydrogen peroxide catalysed by lactate oxidase triggering the degradation of arylboronate gating fragments, resulting in the opening of the NPs and thus controlled drug release [183].

#### 4.3.2. Tumour-Responsive Nanocarriers in Chemoimmunotherapy

Although conventional chemotherapy is not classed as immunotherapy, several chemotherapeutic agents, including oxaliplatin, doxorubicin and paclitaxel, can stimulate immunogenic cell death (ICD). This process promotes the release of damage-associated molecular patterns, which activate antitumour immune responses. However, when administered systemically at high doses, these agents often suppress the immune system, limiting their ability to trigger sustained antitumour immunity [21]. To overcome this limitation, tumour-responsive nanocarriers have been developed to deliver chemotherapeutic drugs more selectively to the TME. These nanosystems are typically designed to respond to specific tumour-associated stimuli, such as acidic pH or elevated ROS levels, enabling controlled drug release at the target site while minimising off-target toxicity. For example, dual pH/ROS-sensitive nanoparticles co-loaded with chemotherapeutics like docetaxel and doxorubicin have demonstrated enhanced tumour accumulation and improved ICD induction in preclinical models [21,77]. Moreover, combining chemotherapeutics with immunomodulatory agents within these nanocarriers has shown promise in amplifying immune responses against tumours. This dual-delivery approach not only enhances direct cytotoxicity but also promotes immune cell recruitment and activation within the TME [184]. While these strategies have yielded encouraging results in animal studies, translating them into clinical practice remains challenging. Further research is needed to refine nanocarrier designs, ensure precise targeting and confirm safety and efficacy in human trials.

#### 4.3.3. pH Sensitivity

Modifying the release kinetics of the NP cargo is an essential aspect for overcoming MDR, as it ensures that the encapsulated drug is delivered effectively within the TME, minimising systemic toxicity and maximising efficacy [76]. Stimuli-responsive NPs are engineered to release their cargos in a controlled manner in response to physicochemical or biochemical stimuli such as temperature and pH, by undergoing a phase transition to increase the permeability of their membranes [133]. Among the diverse stimuli exploited for NP active targeting, pH change has received considerable attention due to the presence of multiple pH gradients within the body. The TME is typically more acidic (pH ~6.8) compared to the extracellular pH of healthy tissues (pH 7.4) in both epithelial and non-epithelial tumours. This acidity primarily results from both oxidative phosphorylation and anaerobic glycolysis in hypoxic conditions, which leads to the accumulation of lactic acid [126,185]. To capitalise on this difference, a pH-sensitive nanoliposomal formulation of doxorubicin was developed for glioma treatment. This system remains stable at physiological pH but releases the encapsulated drug selectively within acidic tumour tissues [83,186,187]. The pH-responsiveness and stability of this system were further confirmed through the incorporation of paclitaxel into the liposomal bilayer of a PSL. At a physiological pH of 7.4, the liposomes demonstrated stability, while alterations in the supramolecular structure were noted at a lower pH, highlighting their pH-sensitive behaviour [188]. Furthermore, a recent study employing a daunorubicin liposomal formulation enriched with Cardiolipin validated its high drug encapsulation efficiency, rapid release profile in low pH and notably enhanced cytotoxicity compared to both daunorubicin alone and liposomes resembling DaunoXome^®^ [189].

The application of PSLs as drug carriers offers significant advantages, facilitating site-specific drug release while minimising systemic toxicity and improving therapeutic efficacy by reducing off-target drug distribution. For instance, a study using irinotecan, commonly employed for the treatment of various cancers including colorectal cancer, utilised folate-coated PSLs as its drug delivery system. The findings demonstrated prolonged and sustained release of the encapsulated drug in a pH-dependent manner, which reduced premature drug leakage at physiological pH [161]. Notably, the liposomal formulation exhibited a particle size below 200 nm, indirectly demonstrating the potential for enhanced tumour accumulation via the EPR effect [161]. Moreover, the study reported significant improvements in the antitumour activity with no notable increase in toxicity when compared to irinotecan alone [161]. Similarly, reduced toxicity has been observed with liposomal formulations of doxorubicin. In one study, mice administered with intravenous doxorubicin-loaded PSLs experienced decreased hepatic injury and reduced renal toxicity compared to those receiving free doxorubicin [190].

Collectively, these findings underscore the potential of PSL formulations in enhancing drug delivery and treatment efficacy, while minimising adverse effects, highlighting their promise in cancer therapeutics. Continued optimisation and clinical translation of such systems could play a pivotal role in advancing precision medicine strategies to overcome MDR in cancer treatment.

#### 4.3.4. Enzyme Responsiveness

Enzyme-responsive NPs leverage the aberrant expression of enzymes in the TME such as matrix metalloproteinases (MMPs) or cathepsins (e.g., cathepsin B) [175,191]. These enzymes cleave peptide linkers on the NP surface and trigger site-specific drug release. MMP-cleavable peptide-coated micelles loaded with paclitaxel demonstrated superior tumour penetration and therapeutic efficacy in resistant breast cancer models [192]. In another study, MMP-2/9-sensitive micelles demonstrated improved tumour targeting and accumulation compared to nonsensitive micelles in vivo, due to specific release of cargo at the tumour site. The in vivo data demonstrated that MMP-2/9-responsive micelles effectively suppressed solid tumours with low systemic toxicity [193].

#### 4.3.5. Redox Responsiveness

Tumour cells are known to exhibit high intracellular levels of GSH especially in drug-resistant phenotypes [194]. Redox-responsive NPs are engineered with disulphide bonds within the structure that remain stable extracellularly but are cleaved in the reductive intracellular environment. This triggers the release of encapsulated cargo such as doxorubicin or siRNA, directly into the cytoplasm, allowing the bypass of drug efflux transporters. This is a selective intracellular activation that can significantly improve the therapeutic index as well as overcoming one of the key resistance mechanisms in MDR cancers [194,195].

#### 4.3.6. Hypoxia Responsiveness

Tumour hypoxia is a hallmark of solid tumours and plays a pivotal role in cancer resistance by promoting angiogenesis via the HIF-1α pathway, driving epithelial−mesenchymal transition, invasion, metastasis and stem-like traits, as well as enhancing glycolysis [196]. The prevalence of hypoxia in solid tumours and its role in treatment resistance make it an ideal trigger for targeted drug delivery [197]. Hypoxia-responsive NPs are designed using moieties like nitroimidazole, nitrobenzyl alcohols and azo compounds mainly, and these can induce significant changes in the hydrophobicity and surface charge of NPs under hypoxic conditions, thereby altering their behaviour such as structural destabilisation [25]. Several hypoxia-responsive NPs have been developed so far. Although none have yet reached clinical approval, many have demonstrated strong potential in preclinical models [196,198,199,200]. For example, hypoxia-responsive polymer micelles encapsulating both dicoumarol (NAD(P)H:quinone oxidoreductase 1 inhibitor) and sorafenib (anticancer agent) were found to repress the expression of HIF-1α, which significantly sensitised the ovarian cancer cell line to sorafenib-induced apoptosis [201]. By enabling selective release within hypoxic tumour regions—often associated with drug resistance—these nanocarriers offer a promising approach to overcome therapeutic barriers. Delivering therapeutic agents into the resistant areas can aid in re-sensitising of the tumours to chemotherapy.

### 4.4. Stability and Reproducibility Challenges

Multifunctional NP systems, which often combine therapeutic cargos, targeting ligands, imaging agents and stimuli-responsive components, pose notable challenges in terms of long-term stability and reproducibility. Interactions between these components can lead to aggregation, phase separation during storage or even premature drug release. mRNA-loaded LNPs formulated with ionizable lipids, for example, are prone to degradation at refrigerated temperatures due to hydrolysis of lipids and reactive aldehydes forming, which can impair its function. It was recently demonstrated that piperidine-based lipids reduce these degradants, allowing mRNA-LNPs to remain more stable and active for at least five months at 4 °C [202]. Similarly, another study showed that lyophilisation of LNPs with 8% sucrose can help preserve the physical and functional properties upon reconstitution [203]. However, despite these advances, scaling up these systems remain a challenge. Significant variability in particle characteristics can occur by small variations such as flow rates or excipient ratios. To tackle this, Quality by Design principles and real-time process monitoring technologies are being implemented increasingly, to achieve consistent, stable and clinically viable NP products [204].

### 4.5. Combination and Co-Delivery Approaches

The co-delivery of multiple therapeutic agents within a single NP system has been shown to enhance the synergistic anticancer effects as well as reduce the chance of developing drug resistance [205]. Although co-delivery NP systems are not yet clinically approved, several promising preclinical studies have demonstrated their potential in enhancing cancer therapy. Notably, co-encapsulation of anticancer drugs and efflux pump inhibitors, or with gene-silencing agents (e.g., siRNA) using NPs such as nanoliposomes, has demonstrated enhanced efficacy in MDR cancer models [78,205]. Co-delivery of paclitaxel with MDR1-targeting siRNA using biodegradable polymeric NPs significantly increased the cytotoxic effect of paclitaxel in drug-resistant ovarian cancer cells, effectively restoring sensitivity to a level comparable with drug-sensitive cells [206].

In a recent study, PEGylated PSLs were co-encapsulated with gemcitabine and curcumin, as an MRP5 inhibitor, for gemcitabine-resistant PDAC. Gemcitabine was loaded in the aqueous core of the liposome, while curcumin was loaded in the bilayers. The co-treatment with curcumin enhanced the intracellular accumulation of gemcitabine in the MIA PaCa-2 cells in a concentration-dependent manner, as well as synergistic cytotoxicity. In pharmacokinetic studies, a 3- to 4-fold increase in the area under the concentration−time curve was observed, in addition to a significant decrease in plasma clearance and volume of distribution for both drugs. The plasma concentration of gemcitabine was also increased, potentially via inhibition of MRP5 [205]. Likewise, co-encapsulation of paclitaxel with lapatinib (a HER2-TKI) in a polymeric nanocapsule formulation resulted in greater inhibition of MDR ovarian cancer cell growth compared to paclitaxel alone [207].

Micelles co-loading doxorubicin and lapatinib demonstrated suppression of P-gp in resistant breast cancer cell lines and significantly enhanced the in vitro and in vivo antitumour activity [208]. Similarly, a study reported the design of fucoidan−silica−carbon nano-onion NPs (FSCNO) to co-deliver a P-gp inhibitor, HM30181A and doxorubicin specifically to tumour vasculature. This approach minimised drug accumulation in normal tissues but enhanced doxorubicin retention in the resistant cancer cells, leading to effective tumour cell destruction. The FSCNO-doxorubicin-H formulation showed a 3–4 fold increase in doxorubicin nuclear localisation in MDR cancer cells compared to FSCNO loaded with doxorubicin alone [209]. Additionally, lipid−saporin NPs were utilised to bypass efflux transporters via co-delivery of anticancer drugs with ABC transporters, which led to an increase in intracellular drug concentrations and re-sensitisation of drug-resistant cancer cells to chemotherapy [210].

Beyond their pharmacokinetic-modulating properties, NPs can also enhance the host immune response and overcome resistance to cancer immunotherapy. Resistance to immune checkpoint inhibitors (ICIs), particularly anti-PD-1/PD-L1 therapies, remains a significant challenge in current cancer immunotherapy [211]. A notable example of reducing ICI resistance is the use of LNPs carrying STING agonists, which stimulate liver macrophages to produce type I interferons, thereby activating NK cells and enhancing the efficacy of ICIs [123]. Similarly, a combination of ICIs and immuno-NPs co-encapsulating a STING and TLR4 agonist resulted in significant efficacy, curative responses and protective immunological memory in mouse models of breast cancer or melanoma [212]. The immunosuppressive tumour microenvironment also plays a critical role in conferring ICI resistance. A recent review has summarised various nanomedicines targeting components of the tumour microenvironment to enhance the efficacy of ICIs [213]. While numerous combination therapies or co-delivery systems have shown promise in preclinical models, clinical translation remains limited.

### 4.6. Cancer Types Showing Promise with NP-Based Approaches

NP-based therapies have shown encouraging results in specific cancer types where drug resistance presents a major clinical challenge. In breast cancer, particularly triple-negative and HER2-positive subtypes, multiple nanosystems have been designed to overcome P-gp-mediated drug efflux. For instance, polymeric NPs co-delivering doxorubicin and P-gp inhibitors have enhanced cytotoxicity in triple-negative breast cancer stem cells while reducing non-specific uptake in normal tissues [214]. Similarly, mesoporous silica NPs co-loaded with paclitaxel and quercetin have successfully re-sensitised resistant MCF-7/ADR cells [215,216]. In ovarian cancer, preclinical studies demonstrate that gold NPs suppress cisplatin-induced EMT and stemness in A2780 and SKOV3 models, reducing *MDR1/ABCG2* expression and sensitising tumours to platinum chemotherapy [217].

PDAC remains challenging due to dense stroma and poor perfusion. In a recent Phase I/IIa “Carolyn Trial”, EGFR-targeted nanocells loaded with irinotecan exhibited disease stabilization in up to 40% of heavily pre-treated patients, with manageable toxicity profiles [218]. Preclinically, pathophysiology-driven NPs designed to penetrate dense stroma and release their payloads in response to pancreatic tumour enzymes demonstrated >2-fold improvement in gemcitabine uptake and doubled median survival in orthotopic mousse models [219].

## 5. Advanced Nanoparticle Strategies

### 5.1. Gene-Directed NP Strategies to Overcome MDR

Utilising NPs as delivery vehicles for nucleic acid-based therapies, including siRNA, miRNA, antisense oligonucleotides and CRISPR/Cas9 systems, represents an emerging and highly promising strategy to combat MDR in cancer therapy. These gene-based approaches enable the selective silencing or permanent modification of key resistance-related genes, such as those encoding ABC transporters, anti-apoptotic proteins (e.g., BCL2) and DNA repair enzymes [220]. The clinical application of naked nucleic acids is limited due to their poor stability, rapid degradation in circulation and inefficient cellular uptake [221,222]. NP platforms can protect nucleic acids from enzymatic degradation, enhance their pharmacokinetic profiles and facilitate tumour-targeted delivery through passive (e.g., EPR) or active (ligand-mediated) mechanisms. These systems can also be engineered to respond to tumour-specific stimuli, ensuring controlled release and, thus, effective gene modulation.

Recent studies have demonstrated the efficacy of NP-mediated co-delivery of chemotherapeutic agents with gene editing components to overcome MDR. For instance, a trastuzumab-conjugated liposomal formulation was engineered to co-encapsulate paclitaxel and anti-*ABCB1* siRNA, to enhance cytotoxicity against HER2-positive breast cancer cells by silencing *ABCB1*, the gene encoding P-gp [223]. This system demonstrated a 4.19-fold lower IC_50_ value compared to the unencapsulated paclitaxel (PTX) and a 2.82-fold lower IC_50_ value compared to liposomes without trastuzumab (PTX-SL). They also exhibited 2.67- and 1.38-fold greater retention of paclitaxel at the tumour tissues 6 h post injection relative to PTX and PTX-SL, respectively. Moreover, there was a significant decrease in the tumour volume in mice as well as a significant reduction in the expression of HER2, ABCB1 and BCL2 markers in both in vitro and in vivo samples [223]. Similarly, folate-decorated PEGylated cationic triblock copolymer was chemically conjugated with both doxorubicin and BCL2 siRNA. It was engineered to be released in a reduction and pH dual-sensitive manner, in which the release of the cargoes was shown to enhance significantly in a reductive and acidic environment mimicking those of cancer cells’ endosomes and lysosomes [224]. This combination demonstrated efficient co-delivery of doxorubicin and BCL2 siRNA into MCF-7 cells and enhanced cell apoptosis and anticancer effects [224]. Furthermore, a pH- and redox-responsive (ssPalm) LNP encapsulating mRNA achieved 95.0 ± 2.1% transfection efficiency in human brain capillary endothelial cells. Moreover, no detectable cytotoxicity was observed at 6.25 μg/mL, whereas Lipofectamine induced morphological changes and cell detachment [225]. Notably, the ssPalm-LNPs disturbed the expression of 40 proteins, which was significantly lower compared to Lipofectamine (84 proteins), as revealed by the proteomic SWATH-MS analysis [225].

**Figure 6:**
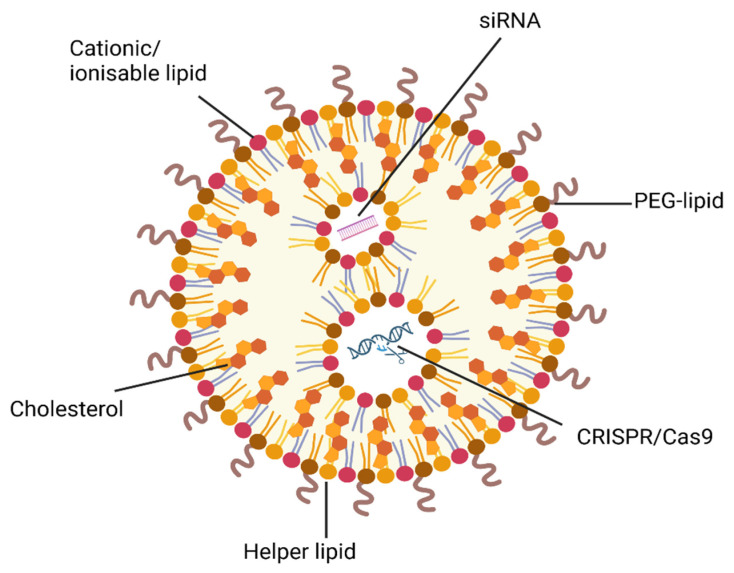
LNP formulation for CRISPR/Cas9 and siRNA delivery. This figure was created in BioRender (Park, S. (2025); https://BioRender.com/4njo55w, accessed on 13 May 2025).

In the realm of genome editing, LNPs delivering gene editing cargos (e.g., CRISPR/Cas systems) have achieved remarkable in vivo editing efficiencies (Figure 6) [226]. A wide variety of LNP systems can be utilised for the delivery of CRISPR/Cas9 genome editing. A study has demonstrated efficient delivery of Cas9 mRNA and sgRNAs via LNPs into orthotopic glioblastoma, achieving up to 70% gene editing in vivo. This led to apoptosis of the cancer cell and up to 50% tumour growth inhibition, highlighting the therapeutic potential of LNP-mediated CRISPR delivery in aggressive tumours [227]. More recently, a study demonstrated that optimised LNPs encapsulating CRISPR base or prime editor ribonucleoproteins (RNPs) can further enhance genome editing outcomes, achieving improvements of over 300-fold in in vivo editing efficiency compared to unencapsulated RNPs [228]. Using ionisable lipids such as SM102 and optimising PEG-lipid content, these chemically defined LNPs provide potent and safe editing opportunities with minimised off-target effects. Moreover, the LNPs demonstrated enhanced on-target editing efficiency and lower bystander editing compared to unformulated RNPs. In vivo, this led to functional protein restoration, underscoring their potential as a promising strategy for the future of transient, non-viral gene therapy [228].

Optimised delivery of CRISPR/Cas systems and siRNA using NPs offers new avenues to disrupt genes like *ABCB1*, *ABCC2* or *BCL2* and overcome MDR. It opens the gateway to utilising gene editing for the treatment of many clinical diseases and developing targeted or personalised therapies with intracellular mRNA delivery via LNPs to achieve cell-selective CRISPR/Cas9 genome editing. There are currently many siRNA- and CRISPR/Cas9-NP systems undergoing clinical trials (Table 6). However, improved targeting, editing precision and minimisation of immune responses will be crucial for safe, in vivo gene therapy.

To provide a practical synthesis of the above strategies, Table 7 maps a few specific NP design features to the MDR mechanisms they aim to overcome, along with recent representative examples.

### 5.2. Safety and Ethical Considerations for Gene Editing Nanomedicines

While NP platforms can significantly enhance the efficiency of CRISPR/Cas9 delivery, several safety and ethical challenges must be addressed before clinical application.

#### 5.2.1. Off-Target Gene Editing

Off-target cleavage at genomic sites with only partial sgRNA complementarity may potentially cause harmful mutations, chromosomal rearrangements or even oncogene activation [236]. High-throughput assays such as GUIDE-seq and CIRCLE-seq have shown that, despite improvements in Cas9 specificity, low-frequency off-target events continue to occur in vivo. This highlights the need for the development of ultra-high-fidelity nuclease variants and application of rigorous gRNA design pipelines to keep unwanted edits to a minimum [236].

#### 5.2.2. Immunogenicity of CRISPR Components

Cas9 enzymes derived from *S. pyogenes* are recognized by pre-existing immunity in up to 80% of adults. Both the nuclease and repeated NP doses can trigger innate and adaptive immune responses, potentially reducing editing efficiency and increasing the risk of inflammation or hypersensitivity reactions [237]. To address these challenges, several approaches have been explored, including transient delivery of Cas9-sgRNA RNP complexes, humanized or orthogonal nuclease variants and “stealth” NP coatings designed to attenuate immune recognition [237].

#### 5.2.3. Ethical and Societal Implications

CRISPR/Cas9 therapies raise crucial ethical questions even in non-germline (somatic) contexts around informed consent for irreversible genomic alterations, equitable access to advanced therapies and long-term monitoring for unexpected effects [238]. There are also on-going debates around the concern that NP-mediated ease of delivery could blur the lines between treatment and enhancement purposes. Experts have recently highlighted the need for strong regulatory frameworks, transparent patient education and broad public engagement to ensure responsible development and prevent the misuse of these systems for either “designer” traits or unapproved enhancement applications [238].

### 5.3. Extracellular Vesicles: Opportunities and Limitations

In addition to synthetic NP platforms, extracellular vesicles (EVs) have also emerged as promising endogenous carriers for therapeutic delivery. EVs, including exosomes and microvesicles, offer natural advantages such as low immunogenicity, intrinsic targeting ability and the ability to transport bioactive cargos like siRNA, miRNA or chemotherapeutic drugs [239]. Recent studies show that EVs can also contribute to overcome MDR by facilitating the delivery of functional therapeutic cargos such as siRNA, miRNA and small drugs directly into tumour cells, thereby modulating gene expression or bypassing efflux transporter-mediated drug expulsion [240]. However, the clinical translation of EVs is also hindered due to limited scalability, inconsistent drug loading, batch heterogeneity and unclear pharmacokinetics. Contrarily, synthetic nanocarriers often offer more tunable properties, reproducibility and established manufacturing pipelines [239,240].

## 6. Translational and Clinical Considerations

### 6.1. Manufacturing Scalability and Reproducibility

When transitioning NP production from laboratory-scale setups to commercial manufacturing, batch-to-batch inconsistencies often emerge. Key quality attributes such as particle size, composition and encapsulation efficiency must be tightly regulated, as deviations in any of these parameters can undermine both safety and therapeutic performance. Liposomal formulations, in particular, are prone to heterogeneity, and without careful control, variable pharmacokinetics and therapeutic outcomes can be expected [241]. Employing advanced tools such as real-time nuclear magnetic resonance or inline liquid chromatography−mass spectrometry, integrated with continuous microfluidic platforms or high-throughput screening, has demonstrated substantial improvements in product consistency and enables rapid detection and correction of potential deviations during large-scale manufacturing [241]. Nevertheless, these approaches incur additional complexity and costs, requiring significant upfront investment and multidisciplinary expertise to establish workflows compliant with good manufacturing practice standards.

### 6.2. Regulatory Pathways and Quality by Design

Regulatory classification of nanomedicines is often difficult due to their hybrid nature, straddling the line between small-molecule drugs and biologics as an interdisciplinary field [242]. As of now, there is no single global standard for defining or testing materials at the nanoscale, so agencies like the FDA and the European Medicines Agency often require different data [242]. For successful translation from bench to bedside, early engagement with regulatory bodies is needed to define critical quality attributes, establish safety and immunotoxicity assays, and agree on the pharmacokinetic and dosimetry assessment methods [243]. Meanwhile, initiatives at both national and international are making efforts to streamline testing and reporting. The “Assay Cascade Protocols” and both the U.S. and Europe Nanotechnology Characterization Laboratories offer stepwise workflows for physicochemical and biological evaluation of nanomaterials. By promoting standardised methods and transparent data-sharing standards, these programs help reduce uncertainty and streamline regulatory assessments [242].

Notably, two major trends are rising to reshape how nanomedicines may be assessed and regulated. Artificial intelligence (AI)-driven tools are increasingly being used to analyse large nanotoxicology datasets and pinpoint safety- and efficacy-critical parameters, enabling “safer-by-design” development, though improved model transparency is needed before AI is fully embraced by regulators [242]. At the same time, environmental impact is gaining regulatory attention. Green-nanotechnology strategies such as biodegradable materials, energy-efficient processes and waste-reduced synthesis, alongside mandatory environmental risk assessments, are becoming essential to demonstrate both safety and sustainability [242].

### 6.3. Patient Heterogeneity and Companion Diagnostics

Tumour uptake of NPs via the EPR effect can differ dramatically both between patients and across lesions in the same patient, making fixed-dose regimens inherently unpredictable [244]. Recent advances focus on integrating imaging-guided companion diagnostics (e.g., dynamic contrast enhanced MRI) to noninvasively measure vascular permeability and predict tumour uptake of NPs. In an early-phase study, a positron emission tomography-visible liposomal probe stratified patients into “high” and “low” uptake groups, promoting tailored dosing and improved response rates [245].

Furthermore, adaptive trial designs are being piloted now that use real-time imaging feedback to refine patient stratification and synchronise multiple, smaller NP doses with peaks in vascular permeability rather than fixed intervals. When paired with molecular biomarkers such as circulating exosomal signatures, this strategy advances further towards personalised nanomedicine, optimising tumour targeting while minimising off-target effects [246].

### 6.4. Clinical Evaluation and Key Translational Barriers

Over the past decade, several NP platforms have advanced into clinical trials or even commercialisation for oncology indications, yet each has exhibited distinct hurdles to broader application. Liposomal formulations like Doxil^®^ and Onivyde^®^ demonstrated the benefits of prolonged circulation and reduced off-target toxicity, but limitations associated with maintaining tight size control and uniform drug loading at scale were seen [89,247]. Albumin-bound particles such as Abraxane^®^ exploited natural gp60/Caveolin-1 transport to boost tumour delivery but prompted hypersensitivity reactions that necessitated premedication and complex regulatory scrutiny [248]. LNPs for nucleic acids, exemplified by the FDA-approved siRNA therapy Onpattro^®^ (patisiran), underscored the importance of defining critical quality attributes for ionisable lipids, managing complement-related pseudoallergy and ensuring cold-chain stability [249,250]. In addition, in early-phase trials of CRISPR/Cas9 LNPs for liver disorders, researchers already face dose-limiting cytokine release, strict off-target surveillance and tailored consent processes for irreversible genome edits [251,252].

## 7. Conclusions

NP-based drug delivery strategies offer a promising and multifaceted approach to overcoming MDR in cancer therapy. Nanocarriers can address many limitations and challenges associated with conventional chemotherapy by enhancing the intracellular drug accumulation at tumour sites, bypassing efflux transporters, facilitating targeted delivery and even enabling in vivo gene modulation. Stimuli-responsive systems, active targeting ligands and co-delivery of gene editors or efflux pump inhibitors have shown great potential in preclinical studies to improve therapeutic outcomes and reduce off-target toxicity. However, challenges remain, particularly in relation to tumour heterogeneity, biological barriers in the TME, immunogenicity and variability in NP uptake, which limit clinical translation. Moreover, the complexity of designing multifunctional nanocarriers that are safe, reproducible, and scalable for clinical use demands further optimisation. Future research should focus on the clinical translation of promising nanosystems that integrates precision medicine approaches and tumour-specific targeting, and advances in non-viral genome editing techniques. Looking forward, the clinical success of NP-based therapies against MDR will depend on optimising delivery specificity, safety and scalability. Advances such as CRISPR-loaded NPs, AI-guided formulation and patient-personalised nanomedicine are emerging rapidly. To bridge the gap between preclinical potential and clinical use, challenges such as tumour heterogeneity, immune clearance and regulatory hurdles must be overcome.

To overcome these limitations, next-generation nanoplatforms should be strategically designed with a strong emphasis on enabling clinical translation. This includes, but is not limited to, the use of clinically approved, biodegradable materials, simplifying formulation procedures for enhanced reproducibility and modular designs that allow precise tuning of features such as the targeting ligands, the release kinetics and immune evasion. Companion diagnostics and image-guided delivery systems can further aid in personalisation of treatments and optimise NP performance. Ultimately, aligning NP platform design with pharmacological, manufacturing and regulatory considerations from the outset will be critical for achieving clinical success in combating MDR.

## Figures and Tables

**Table 1:** Clinically established liposomal drug delivery systems.

Product Name	Drug	Key Feature (s)	Indication (s)	Reference
Doxil^®^/Caelyx^®^	Doxorubicin	PEGylated, prolonged circulation and enhanced tumour accumulation.	Kaposi’s sarcoma, ovarian cancer and multiple myeloma	[89]
Myocet^®^	Doxorubicin	Non-PEGylated liposomes and reduced cardiotoxicity.	Metastatic breast cancer (with cyclophosphamide)	[90]
DepoCyt^®^	Cytarabine	Sustained-release liposomes for intrathecal administration, prolonged drug half-life.	Lymphomatous meningitis	[91]
Vyxeos^®^ (CPX-351)	Daunorubicin +Cytarabine	Co-encapsulation of drugs in liposomes, shown synergistic effects and enhanced anti-leukemic activity.	Acute myeloid leukaemia (AML)	[92]
Mepact^®^	Mifamurtide	Less than 100 nm multi-lamellar liposome, postoperative combination with other anti-neoplastic agents has improved survival.	Non-metastatic osteosarcoma	[93]
Onivyde^TM^	Irinotecan	PEGylated, prolonged circulation, enhanced controlled release and enhanced tumour accumulation.	Metastatic pancreatic cancer	[94]
DaunoXome^®^	Daunorubicin	Small (~45–80 nm) unilamellar liposome, reduced cardiotoxicity and improved tolerability.	HIV-related Kaposi’s sarcoma	[95]
Marqibo^®^	Vincristine sulfate	Liposomal vincristine, prolonged circulation and reduced neurotoxicity.	Philadelphia chromosome-negative (Ph-) acute lymphoblastic leukaemia	[96]

**Table 2:** A comparative summary of different NP platforms in cancer therapy.

Platform	Key Feature(s)	Advantages	Limitations	References
Gold NPs (AuNPs)	Tunable size/shape and strong optical/plasmonic properties	Greate for imaging, photothermal therapy and targeted delivery	Potential hepatic accumulation, size/ligand-dependent toxicity and/or limited clinical translation	[101]
LNPs	Ionizable phospholipids, scalable microfluidic formulation	Clinically validated for RNA delivery (e.g., Onpattro^®^ and vaccines) and high encapsulation efficiency	Cold-chain required, hydrolytic instability and immunogenicity risk	[18,102,103]
Polymeric NPs	Biodegradable polymers (e.g., PLGA and micelles, dendrimers)	Controlled/sustained release, multifunctionality and tunable chemistry	Complex synthesis, potential degradation toxicity and scale-up challenges	[18,104,105]
Lipid−polymer hybrid NPs	Core–shell combining polymer and lipid components	Blended benefits: stability, payload versatility and prolonged circulation	Complex formulation, mostly preclinical	[106]
Mesoporous silica NPs	High surface area and easily functionalized, porous structure	High drug loading and stimuli-responsive kinetics	Poor biodegradability and risk of long-term accumulation	[107,108]
Dendrimers	Highly branched, monodisperse synthetic polymers	Precise size control, abundant functional groups and high payload capacity	Potential cytotoxicity at high generation and renal clearance limitations	[18,109,110]
Magnetic (SPIO) NPs	Superparamagnetic iron oxide core	MRI traceability and magnetically guided targeting	Limited penetration depth and aggregation or iron overload risks	[111]
Albumin-based NPs (e.g., nab-paclitaxel)	Protein-based carrier (e.g., albumin)	Clinically approved, endogenous uptake pathways, biocompatible	Hypersensitivity risk and variable manufacturing batch quality	[112,113]

**Table 3:** Nanoparticle design features that enhance the EPR effect.

NP Design Feature	Contribution Towards the EPR Effect
Size (typically 50–200 nm)	Small enough to pass through the leaky tumour vasculature and large enough to avoid renal clearance.
Surface charge	Neutral or slightly negative/positive surfaces reduce opsonisation and RES (reticuloendothelial system) clearance, improving circulation time and tumour accumulation.
Hydrophilic coatings (e.g., PEGylation)	Increases blood circulation half-life by preventing protein adsorption and immune recognition—more time to accumulate via EPR.
Shape (e.g., spherical)	Influences margination, internalisation and clearance rates—improving tumour delivery.

**Table 4:** Different active targeting nanosystems for cancer therapy.

Type of NP	Targeting Ligand	Drug	Cancer Types	Reference
Liposomes (Active Targeting)	Anti-HER2 monoclonal antibodies (e.g., Trastuzumab)	Doxorubicin	HER2-positive breast cancer	[136]
Gold NPs (AuNPs)	Estrogen receptor ligands	Doxorubicin	Breast cancer	[137]
Transferrin-conjugated NPs	Transferrin	Doxorubicin	Various cancers	[138,139]
Folate-conjugated liposomes	Folate receptor	Doxorubicin and Methotrexate	Ovarian, colon cancer and breast cancer	[140,141]
Aptamer-conjugated NPs	PD-L1, PSMA and CD44	Doxorubicin and siRNA	Prostate cancer, lung cancer and glioblastoma	[142,143,144,145]
Liposomes (pH-responsive, active targeting)	pH-sensitive ligands (e.g., RGD peptide)	Paclitaxel and Doxorubicin	Ovarian cancer and breast cancer	[146,147,148]
Polymeric NPs	Anti-EGFR and Anti-CD20	Doxorubicin and Paclitaxel	Glioblastoma, lymphoma and colon cancer	[149,150,151]
Mesoporous silica NPs	Folate and HER2 antibodies	Paclitaxel and Doxorubicin	Ovarian cancer and breast cancer	[152,153,154,155,156]
Folate- and biotin- conjugated liposomes	Folate and biotin receptors	Methylnaphthazarin	Cervical cancer	[157]
Quantum dots	Anti-EGFR and Anti-HER2	Doxorubicin and siRNA	Prostate, breast and lung cancer	[158,159]

**Table 5:** Stimuli-responsive nanoparticle strategies.

Stimulus Type	Trigger Mechanism	Advantages	Role in MDR	Reference
Enzyme-responsive	Overexpressed tumour enzymes (e.g., MMPs, cathepsins) degrade linkers/coatings	High tumour specificity and improved penetration	Enhancing accumulation and penetration	[175]
Redox-responsive (GSH)	High intracellular GSH accelerates cleavage of disulfide bonds	Selective intracellular release and bypasses MDR efflux	Bypassing efflux via cytoplasmic-triggered release	[176,177]
Hypoxia-responsive	Hypoxia-induced enzymes reduce nitro/azo groups or activate prodrugs	Targets resistant hypoxic cores and enhanced selectivity	Delivering drugs into resistant hypoxic zones	[178]
Magnetic-responsive	External magnetic field guides particles and induces hyperthermia	Precise guidance of NPs and combinatorial thermal therapy	Inducing magnetic hyperthermia and improved delivery	[77,160]
Light-responsive (NIR)	NIR light cleaves photo-labile linkers or activates photothermal/photosensitizers	Precise spatial and temporal control	Disrupting tumour survival via local heat or ROS	[179,180]
Ultrasound-responsive	Ultrasound causes cavitation or vaporization triggering release	Non-invasive, site-specific burst release	Boosting penetration and microbubble-assisted uptake	[181,182]

**Table 6:** CRISPR/Cas9 and siRNA NP systems in clinical trials for cancer or related applications.

System	Type of NP	Cargo	Indication	Status	Reference
CALAA-01	Cyclodextrin-based polymer NP	siRNA targeting RRM2	Solid tumours	Phase I completed	[229]
Atu027	Liposomal NP	siRNA targeting PKN3	Metastatic pancreatic cancer	Phase I completed, in combination with gemcitabine	[230]
iPsiRNA (NCT00672542)	Liposomal NP	siRNA targeting immunoproteasome subunits	Metastatic melanoma	Phase I completed	[231]
PD-1 knockout T Cells	Gene-edited T cells (ex vivo, non-viral)	CRISPR-Cas9 PD-1 knockout	Non-small cell lung cancer	Phase I completed (China)	[232]
UCART19	CRISPR-edited allogeneic CAR-T cells	CRISPR-edited TCR/CD52 deletion	Acute lymphoblastic leukaemia	Phase I dose-escalation cohorts completed	[233]

**Table 7:** NP design strategies mapped to MDR mechanisms in cancer.

Design Strategy	Key Feature (s)	Targeted MDR Mechanism	Example	Reference
Stimuli-responsive release	Dual pH/redox-sensitive linkers	Endosomal entrapment and premature drug efflux	Hyperbranched polymeric NPs that release docetaxel in the acidic/reductive TME, enhancing uptake (100% at 0.5 h) and efficacy	[176]
Active targeting via ligands	Surface conjugation with hyaluronic acid	Low receptor-mediated uptake in MDR cells	Doxorubicin- and selenium-incorporated mesoporous silica NPs coated with hyaluronic acid for Haase-triggered DOX release, demonstrating enhanced tumour cell uptake and efficacy in osteosarcoma models	[234]
Charge-reversal and zwitterion surfaces	pH/enzyme-triggered switch from neutral to cationic surface	Variable tumour uptake and protein corona formation	Acid-responsive charge-reversal DDS that remains “stealth” in blood but flips positive in the TME, improving penetration	[170]
Co-delivery of drug + siRNA/CRISPR	Co-encapsulation of chemotherapeutic + gene-silencing/editing	ABC transporter overexpression and anti-apoptosis	LNPs delivering prime-editor RNPs + chemotherapeutic, boosting in vivo editing >300-fold compared to unencapsulated RNPs	[11]
Biomimetic cell-membrane coatings	Cancer-cell membrane cloak for immune evasion and homotypic binding	Mononuclear phagocyte clearance and poor targeting	Cancer cell membrane-encapsulated NPs that escape macrophages and homotypically target tumours, enhancing circulation	[235]

## Abbreviations

MDR	multidrug resistance
NP	nanoparticle
siRNA	small interfering RNA
CRISPR	clustered regularly interspaced short palindromic repeat
TME	tumour microenvironment
ABC	ATP-binding cassette
P-gp	P-glycoprotein
MRP	multidrug resistance-associated protein
BCRP	breast cancer resistance protein
GI	gastrointestinal
CRC	colorectal cancer
GSH	glutathione
ROS	reactive oxygen species
S1P	sphingosine-1-phosphate
EGFR	epidermal growth factor receptor
HIF-1	hypoxia-inducible factor-1
EPR	enhance permeability and retention
SLN	solid lipid nanoparticles
LNP	lipid nanoparticles
PEG	polyethylene glycol
ATR	active transport and retention
ECM	extracellular matrix
RGD	arginine-glycine-aspartic
PSMA	prostate-specific membrane antigen
Tf	transferrin
MAbs	monoclonal antibodies
PTXNR-TTZ	trastuzumab-conjugated paclitaxel-loaded nanorods
PD-1	programmed death-1
ICD	immunogenic cell death
PSL	pH-sensitive liposome
MMP	matrix metalloproteinases
FSCNO	fucoidan−silica−carbon nano-onion NPs
PTX	unencapsulated paclitaxel
PTX-SL	paclitaxel liposome without trastuzumab
ssPalm	pH- and redox-responsive LNP
RNP	ribonucleoprotein
TKI	tyrosine kinase inhibitor
PDAC	pancreatic ductal adenocarcinoma
